# Enhanced activation of matrix metalloproteinase-9 correlates with the degree of papillary thyroid carcinoma infiltration

**DOI:** 10.3325/cmj.2014.55.128

**Published:** 2014-04

**Authors:** Ilona Marečko, Dubravka Cvejić, Sonja Šelemetjev, Svetlana Paskaš, Svetislav Tatić, Ivan Paunović, Svetlana Savin

**Affiliations:** 1 Institute for the Application of Nuclear Energy – INEP, University of Belgrade, Zemun-Belgrade, Serbia; 2 Institute of Pathology, Medical Faculty, University of Belgrade, Belgrade, Serbia; 3 Center for Endocrine Surgery, Institute of Endocrinology, Diabetes and Diseases of Metabolism, Clinical Center of Serbia, Medical Faculty, University of Belgrade, Belgrade, Serbia

## Abstract

**Aim:**

To determine whether matrix metalloproteinase-9 (MMP-9) may be a useful adjunctive tool for predicting unfavorable biological behavior of papillary thyroid carcinoma (PTC) by evaluating the expression profile and proteolytic activity of MMP-9 in PTC by different techniques and correlating the findings with clinicopathological prognostic factors.

**Methods:**

Immunohistochemical localization of MMP-9 was analyzed with antibodies specific for either total or active MMP-9. Activation ratios of MMP-9 were calculated by quantifying gel zymography bands. Enzymatic activity of MMP-9 was localized by in situ zymography after inhibiting MMP-2 activity.

**Results:**

Immunostaining of total and active MMP-9 was observed in tumor tissue and occasionally in non-neoplastic epithelium. Only active MMP-9 was significantly associated with extrathyroid invasion, lymph-node metastasis, and the degree of tumor infiltration (*P* < 0.001, *P* = 0.004, and *P* < 0.001, respectively). Gelatin zymography revealed a correlation between the MMP-9 activation ratio and nodal involvement, extrathyroid invasion, and the degree of tumor infiltration. In situ zymography showed that gelatinases exerted their activity in tumor parenchymal and stromal cells. Moreover, after application of MMP-2 inhibitor, the remaining gelatinase activity, corresponding to MMP-9, was highest in cancers with the most advanced degree of tumor infiltration.

**Conclusions:**

This is the first report suggesting that the evaluation of active MMP-9 by immunohistochemistry and determination of its activation ratio by gelatin zymography may be a useful adjunct to the known clinicopathological factors in predicting tumor behavior. Most important, in situ zimography with an MMP-2 inhibitor for the first time demonstrated a strong impact of MMP-9 activity on the degree of tumor infiltration during PTC progression.

Papillary thyroid carcinoma (PTC) is the most common malignancy of the thyroid, with a rapid global rise in incidence in the recent decades. Despite its generally indolent behavior, a small proportion of PTC patients develop more aggressive forms of the disease. Several clinical and histopathological parameters, such as sex, age, histologic grade, and tumor stage have been reported as useful in improving prognostic stratification (1). However, the prognosis of tumor behavior for individual patients with thyroid cancer can vary greatly, partly due to the marked clinical heterogeneity among such patients, even within a particular histologic group. Therefore, it is important to understand the molecular mechanisms responsible for cancer development, progression, and metastasis, and to establish novel strategies for predicting biological behavior of thyroid cancer and for clinical management of patients.

In cancer research, much interest has been devoted to alterations in expression grade and activity of various matrix metalloproteinases (MMPs) and their corresponding inhibitors (2). MMPs are a family of zinc-dependent endopeptidases with the capacity to degrade extracellular matrix proteins and basement membranes (3), and are therefore strongly implicated in multiple stages of cancer progression. Among the MMPs, a subset called gelatinases, consisting of MMP-2 (gelatinase A) and MMP-9 (gelatinase B), has gained the most attention in studies on the acquisition of invasive and metastatic tumor properties, as they degrade collagen IV, the major component of the basement membrane (4,5). Their proteolytic activity is regulated at various levels, including expression, balance between amounts of enzymes and their inhibitors, pericellular localization, and most importantly, latent form activation. Namely, gelatinases are secreted as inactive proenzymes and are activated after cleavage of the pro-peptide domain of the molecule (6). MMP-9 is of special interest because its basal expression is normally low, whereas it is highly expressed in most human cancers in response to various growth factors and cytokines (7,8). It has been shown that MMP-9-deficient mice exhibit impaired metastasis formation and tumor growth (9). In this respect, up-regulation of MMP-9 expression in various types of human cancers contributes to tumor progression, invasion, and metastasis (10-13).

There is much evidence demonstrating that MMP-9 is overexpressed in various tumor types when compared to normal tissue (14-16). On the other hand, this protein has not been widely investigated in thyroid tumors and there are still some controversies regarding its usefulness as a marker for diagnosis or prognosis of these tumors (17-21). Moreover, the ratio of active-to-total MMP-9 and the precise localization of enzymatic activity in thyroid tissue have not yet been investigated. To localize MMP-9 expression in PTC and corresponding non-tumor tissue, we used two commercial antibodies that recognize either both pro-active and active or only active form of MMP-9 for immunohistochemistry. To our knowledge, active form of MMP-9 has not been immunohistochemically evaluated on thyroid tissue sections previously. We also used gelatin zymography, which is a sensitive, quantifiable assay to analyze pro-active and active form of MMP-9, and sensitive dye-quenched gelatin (DQ-gelatin) in situ zymography with a selective MMP-2 inhibitor to localize the gelatinase activity corresponding to MMP-9 in tissue. Thus, we explored the expression profiles, activation ratio, and localization of MMP-9 activity in tissue sections of papillary thyroid carcinomas and correlated the findings with clinicopathological prognostic factors with the aim of determining whether MMP-9 may be a useful adjunctive tool for predicting unfavorable biological behavior of PTC.

## Material and methods

### Participant selection and clinical data

Thyroid cancer tissues were obtained at thyroidectomy from 120 consecutive patients surgically treated for PTCs at the Center for Endocrine Surgery, Clinical Center of Serbia, Belgrade between 2009 and 2012. The study was approved by the Ethics Committee at the Center for Endocrine Surgery and informed consent was obtained where required.

All specimens were reviewed by a single pathologist, who confirmed the diagnosis of PTC according to the World Health Organization classification of thyroid malignancy (22), identified the histological variant of the tumor, divided fresh tissue into portions, and classified the specimens. A total of 120 routinely processed, formalin-fixed, and paraffin embedded PTC specimens (with adjacent non-tumor tissue, when present) and 32 fresh tissue samples (paired tumor and non-tumor thyroid tissues from the contralateral thyroid lobe) obtained from a cohort of 120 PTC patients were included in this study. All fresh tissue samples obtained immediately after thyroidectomy were snap-frozen in liquid nitrogen and stored at -80 °C to be used for gelatin zymography analysis. At the same time, some of these fresh tissue samples (18 of 32) were fixed in alcohol and embedded in paraffin for evaluation of gelatinolytic activity by DQ in situ zymography.

Formalin-fixed sections from all 120 PTCs were used for immunohistochemical studies. Of these samples, 62 were stained for both total and active MMP-9. The remaining 58 samples were evaluated for total MMP-9 (n = 43) or active MMP-9 (n = 15), giving 105 and 77 samples for total and active MMP-9, respectively.

Before the study, all tissue specimens were reexamined after hematoxylin-eosin staining by a board-certified pathologist for confirmation of the pathologic diagnosis. Moreover, during the histopathological review, 73 cases were subsequently reevaluated for the degree of tumor infiltration, as described previously (23): group A – totally encapsulated tumors (n = 30); group B – non-encapsulated tumors without thyroid capsule invasion (n = 16); group C – tumors with thyroid capsule invasion (n = 10); and group D – tumors with extrathyroid extension (n = 17). Information concerning sex, age, lymph node metastasis involvement, extrathyroid invasion, and tumor size was retrieved by reviewing the pathology reports.

### Tissue extraction

To minimize variation, each pair of thyroid carcinoma and matched normal tissue was extracted and assayed at the same time. Tissue samples were cut into small pieces homogenized in 1 mL of cold extraction buffer (20 mM Tris HCl, 137 mM NaCl, 10% glycerol, 1% nonidet P-40, 2 mM EDTA) and then rotated at 11 000 g for 20 minutes at 4°C in an Eppendorf centrifuge (Eppendorf, Hamburg, Germamy). The supernatant was aliquoted and the protein content was determined using a BCA Protein Assay Kit (Pierce Biotechnology, Rockford, IL, USA).

### Gelatin zymography

The presence of active and latent forms of gelatinases was analyzed in 32 tissue samples by zymography in non-reducing conditions on 8% sodium dodecyl sulfate polyacrylamide gel electrophoresis (SDS-PAGE) containing 1% gelatin. Sample volumes were adjusted to obtain a uniform protein content of 50 µg per lane. After electrophoresis, the gels were washed twice in 2.5% Triton-X and five times in water. The gels were then incubated overnight at 37°C in reaction buffer (5 mM CaCl_2_, and 50 mM Tris-HCl buffer, pH 7.0). Gelatinase activity was visualized by staining the zymograms with Coomassie brilliant blue (0.25% Coomassie brilliant blue G250, 30% acetic acid, 10% methanol, all from Sigma-Aldrich, St Louis, MO, USA) and destaining in acetic acid-methanol-dH_2_O (1:3:6) for 1-2 hours. Gelatinolytic activity was observed as a clear band of digested gelatin against a blue background. To confirm that the visualized zones of lysis were due to MMP activity, selected duplicate gels were incubated using either MMP-2 inhibitor (13.7 nM ARP-100) or 20 mM EDTA.

The MMP-9 activation ratio was calculated from the data obtained by densitometric analysis of the 93 kDa and 83 kDa bands corresponding to latent and active forms of MMP-9, respectively, using TotalLab TL120 software (Nonlinear Dynamics Ltd, Newcastle, UK).

### Immunohistochemistry

A total of 105 PTC sections were stained for total MMP-9, 60 out of which were classified as classical PTC and 45 as the follicular variant (24). Forty-eight classical PTCs and 29 samples of the follicular variant were also stained for activated MMP-9. Immunohistochemistry was performed as described before (25). The sections were incubated with diluted primary antibodies, ie, mouse anti-human MMP-9 activated MCA2736, recognizing the ~ 83 kDa active form of MMP-9 (AbD Serotec, Kidlington, UK) and mouse monoclonal 56-2A4, recognizing both pro-active and active forms of MMP-9 (Abcam, Cambridge, UK). The signal was enhanced with the avidin-biotin-peroxidase complex (Vectastain ABC kit, Vector Laboratories, Burlingame, CA, USA), followed by visualization of the reaction with 3,3′-diaminobenzidine tetrahydrochloride (DAB) solution (Peroxidase Substrate Kit, Vector Laboratories, Burlingame, CA, USA). The slides were counterstained with hematoxylin and examined using an Axio Imager 1.0 microscope (Carl Zeiss, Jena, Germany) with a Canon A640 Digital Camera System (Tokyo, Japan). Negative controls were incubated with PBS in place of the primary antibody and no positive staining was observed.

The tumor sections were examined by two individuals (with no knowledge of the clinical findings) for staining intensity and distribution of immunoreactivity within a single tissue section. Scoring of epithelial staining was evaluated separately for tumor and the surrounding non-tumor tissues. Thus, the staining was graded for total MMP-9 using an empirical semiquantitative system as reported previously (25), in brief: 0) no reaction or focal weak/moderate reaction; 1) strong focal or diffuse weak reaction; 2) moderate diffuse reaction; 3) intense diffuse reaction. The staining for active MMP-9 was graded as follows: 0) no reaction; 1) weak reaction; 2) moderate reaction; 3) intense reaction. Immunohitochemical staining was evaluated by independent observers, with high concordance. When there was doubt between two scores, the observers reexamined the slide together in order to establish a consensus.

### In situ zymography

In situ zymography was performed as described by Hadler-Olsen et al (26). In brief, alcohol-fixed, paraffin-embedded tissue sections were deparaffinized in xylene and rehydrated in graded alcohol baths. Then tissue specimens were washed twice with PBS, overlaid with a solution of 50 μg/mL dye-quenched gelatin (DQ gelatin; Molecular Probes, Thermo Fisher Scientific, Wyman, MA, USA) in reaction buffer and incubated in a dark humidity chamber at 37°C for 2 hours. After that, the sections were rinsed in PBS baths (2 × 5 minutes) and overlaid with DAPI containing antifade solution (S7113, Merck Millipore, Billerica, MA, USA) to counterstain the nuclei. Specimens were fixed with neutral buffered formalin, and green fluorescence indicative of gelatinase activity was observed under a 10 × objective of the fluorescence microscope (Axio Imager 1.0 microscope with AxioCam HR monochrome camera, Carl Zeiss, Jena, Germany).

The level of autofluorescence on the tissue sections was evaluated by substrate incubation on control sections from each tissue at -20°C for 2 hours. To verify the contribution of metalloproteinases, control slides were preincubated with 20 mM EDTA for 1 hour. In addition, to verify the contribution of the two most effective gelatinolytic MMPs, MMP-2 and indirectly MMP-9, slides were coated with substrate containing MMP-2 inhibitor ARP-100 (sc 203522, Santa Cruz Biotechnologies, Santa Cruz, CA, USA) in a final concentration of 13.7 nM. For most of the tissues, at least two independent experiments were performed.

### Statistical analysis

Statistical analysis was performed with the software package SPSS 12.0.1 (SPSS Inc., Chicago, IL, USA) for Windows. The association between immunohistochemical results (positive immunostaining) for each antibody and clinicopathological data was determined using 2-tailed χ^2^ or Fisher exact tests where appropriate, as well as the Spearman rank correlation test. The Mann-Whitney U test was used for comparison between groups, with *P* < 0.05 considered statistically significant.

## Results

### Immunohistochemistry

**Figure 1:**
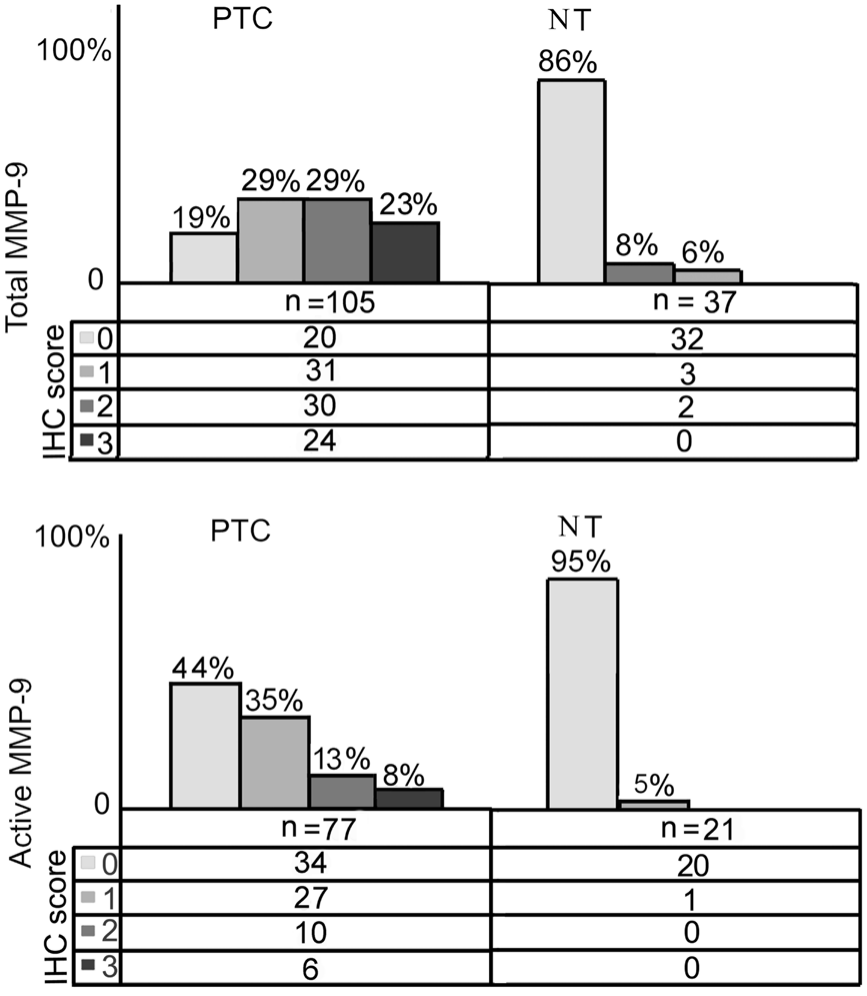
Number and percentage of cases stained for active and total matrix metalloproteinase-9 **(**MMP-9) in papillary thyroid carcinoma (PTC) and nontumor tissues (NT).

Immunohistochemical staining revealed that total MMP-9 was present in the majority of tumors (Figure 1), while the majority of peritumoral tissues remained immunonegative. Active MMP-9 was present in 56% of follicular epithelial cells of PTC cases (Figure 1). When present, the staining was mostly cytoplasmic and sometimes had a granular appearance. In peritumoral tissue, active MMP-9 was detected in only one sample. Positivity for both total and active MMP-9 was also evident to a varying degree in the stromal compartment, inflammatory cells, myo/fibroblast-like cells, and endothelial cells (data not shown).

### Association with clinicopathological parameters

**Figure 2:**
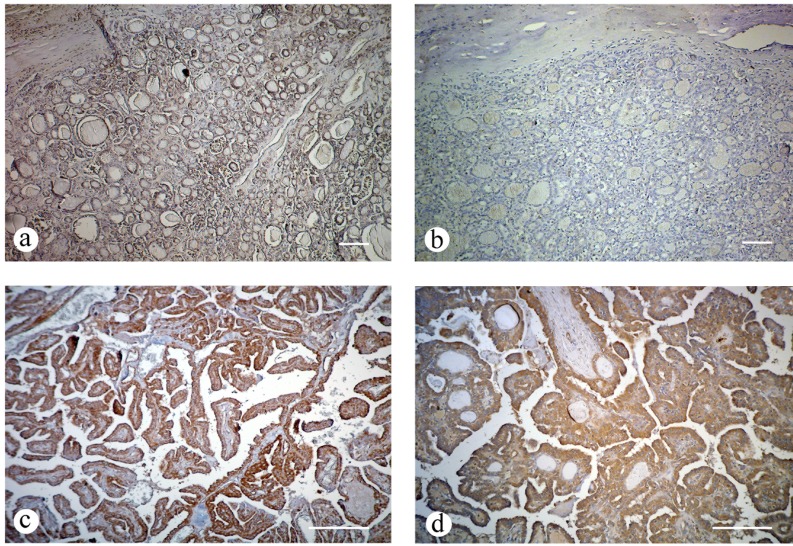
Representative micrographs of immunostaining for total and active matrix metalloproteinase-9 **(**MMP-9) in thyroid tissue samples. (**a**) Moderate diffuse immunoreaction for total MMP-9 in one case of encapsulated papillary thyroid carcinoma (PTC). (**b**) Negative immunostaining for active MMP-9 in the same case. (**c**) Strong diffuse MMP-9 positivity in a case of PTC with extrathyroid invasion. (**d**) Strong staining for active MMP-9 in a case of PTC with extrathyroid invasion.

Immunoexpression of total MMP-9 in PTC samples was not significantly correlated with any clinicopathological feature. On the other hand, immunoexpression of active MMP-9 was positively correlated with lymph node metastases, extrathyroid invasion, and degree of neoplastic infiltration, as indicated both by χ^2^ test (or Fisher exact test when necessary) and Spearman’s correlation analysis (Table 1). A correlation with patient age at diagnosis was shown with Spearman’s analysis, but the χ^2^ test failed to confirm it, and no correlation was found between active MMP-9 and sex or tumor size. An example of total and active MMP-9 immunoexpression in PTCs with different degrees of neoplastic infiltration is given in Figure 2.

**Table 1:** Correlation between total and active matrix metalloproteinase-9 (MMP-9) immunohistochemical expression and clinicopathological features in papillary thyroid carcinoma (PTC) patients*

Clinicopathological characteristic	Total MMP-9				Active MMP-9			
	number of cases		statistical analysis (*P*)		number of cases		statistical analysis (*P*)^‡^	
negative^†^	positive^†^	χ^2^ test/Fisher exact test	Spearman rank correlation test	negative^†^	positive^†^	χ^2^ test /Fisher exact test	Spearman rank correlation test
Age at diagnosis:								
patients <45 y	8	42	0.448	0.144	20	16	0.059	**0.034**
patients ≥45 y	12	43			14	27		
Sex:								
male	6	15	0.214	0.100	5	10	0.347	0.126
female	14	70			29	33		
Tumor size:								
<2 cm	9	27	0.261	0.977	13	10	0.153	0.162
≥2 cm	11	58			21	33		
Lymph node metastasis:								
no	19	67	0.185	0.246	31	31	**0.018**	**0.004**
yes	1	17			2	12		
Extrathyroid invasion:								
no	14	63	0.708	0.067	31	24	**<0.001**	**<0.001**
yes	6	22			1	19		
Degree of neoplastic infiltration:^§^								
A	9	16	0.136	0.100	23	7	**<0.001**	**<0.001**
B	1	11			4	12		
C	2	4			3	7		
D	1	11			2	15		

*Among the patients included in statistics for both total and active MMP-9, one had unknown lymph node metastasis status and was therefore excluded from this category. For active MMP-9, in the category extrathyroid invasion, two samples were excluded because of missing data for tumor invasion. Out of the 73 cases that were reexamined for degree of tumor infiltration (23), 55 were included in the subsequent analyses for total MMP-9 and all 73 reexamined for active MMP-9.

†Immunostainings: negative – score 0; positive – scores 1, 2 and 3.

‡Bolded numbers indicate statistical significance at *P* < 0.05 level.

§Degree of neoplastic infiltration: A – totally encapsulated tumors; B – non-encapsulated tumors without thyroid capsule invasion; C – tumors with thyroid capsule invasion; D – tumors with extrathyroid extension.

### Gel-zymography

**Figure 3:**
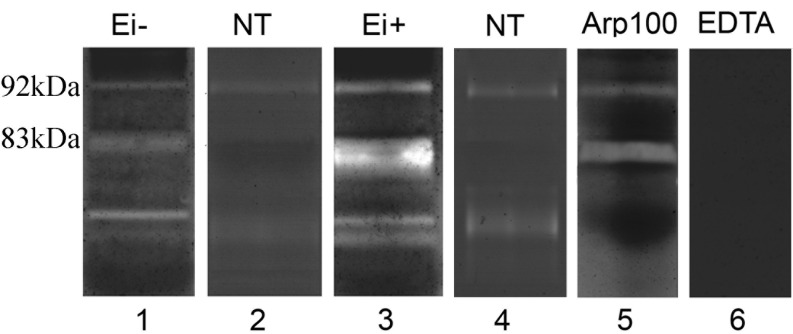
An example of the gelatin zymograms used for matrix metalloproteinase-9 **(**MMP-9) quantitation by densitometry. Gelatin degradation was monitored by sodium dodecyl sulfate (SDS)-8% polyacrylamide gel electrophoresis (PAGE) and Coomassie blue staining. Gelatinolytic bands of 92, 83, 68, and 62 kDa corresponded to proMMP-9, active MMP-9, proMMP-2, and active MMP-2, respectively: NT – non-tumoral thyroid tissue, Ei – extrathyroid invasion. Lane 1, tissue extract of papillary thyroid carcinoma (PTC) sample without extrathyroid invasion (Ei-). Lane 2, paired non-tumor tissue from the same patient. Lanes 3 and 4, tumor and paired non-tumor tissue extract from another PTC sample with extrathyroid invasion (Ei+). Lane 5, a duplicate gel of sample (Ei+) incubated in the presence of 13.7 nM ARP-100, a general inhibitor of MMP-2 activity. Lane 6, the same sample incubated with 10 mM EDTA.

Samples were analyzed for MMP-9 activation by gelatin zymography. Bands corresponding to latent MMP-9 and MMP-2 were clearly detected in all tumor samples, whereas active forms of MMP-9 and MMP-2 showed less distinct bands or no bands in some samples (Figure 3). The levels of MMP-2 and MMP-9 were significantly higher in carcinomas than in peritumoral tissue, irrespective of MMP type or activity state. The specific MMP-2 inhibitor ARP-100 almost completely inhibited MMP-2 on the SDS-gel zymogram, thus confirming its identity, but had almost no effect on MMP-9 in the applied concentration of 13.7 nM. The general metalloproteinase inhibitor, EDTA, completely abolished the reaction in SDS gel zymography (Figure 3). These data verified that these enzymes were all metalloproteinases.

Computer-assisted image analyses of the intensity of proteolytic bands allowed separate quantification of pro-enzyme and active form of the enzyme. These results were used to calculate the ratio of active-to-total MMP-9 for each sample and to correlate it with clinicopathological characteristics of PTC. The activation ratio of MMP-9 was significantly higher in PTC samples presenting with extrathyroid invasion and lymph node metastases than in those without them (*P* < 0.001 and *P* = 0.006, respectively).

**Figure 4:**
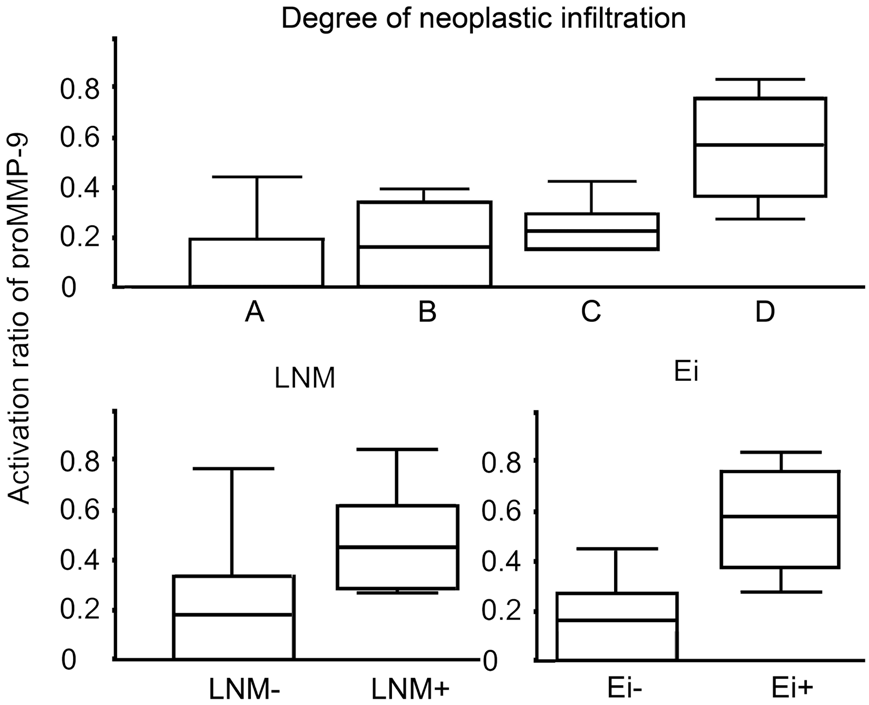
Activation ratio of promatrix metalloproteinase-9 **(**MMP-9) in papillary thyroid carcinoma (PTC) measured by densitometric analysis of zymographic gels. The activation ratios of MMP-9 (proMMP-9) were calculated by dividing the density of the band for active MMP-9 by the sum of the density of the bands for both latent and active forms. The boxes represent the interquartile range and vertical lines show the range of observations. The midlines of boxes give the median values of activation ratios. A, B, C, D are groups of PTC patients with the following degrees of neoplastic infiltration: A – totally encapsulated carcinomas; B – non-encapsulated tumors without thyroid capsule invasion; C – tumors with thyroid capsule invasion; D – tumors with extrathyroid invasion. LNM+ are carcinomas with lymph node metastasis, LNM – tumors without metastases; Ei + and Ei- are tumors with and without extrathyroid invasion, respectively.

When we associated the level of MMP-9 activation with the degree of neoplastic infiltration, a significant difference was found between the activation ratio of the fourth group (D) and any of other three, in other words, when group D was compared to group A, group B, or group C, respectively (*P* = 0.001, *P* = 0.008, and *P* = 0.008) (Figure 4). In addition, Spearman analysis revealed a positive correlation between the activation levels of MMP-9 and the degree of neoplastic infiltration (*P* < 0.001, rs = 0.624). However, there was no significant association with the age of patients or tumor size (data not shown).

When we analyzed the correlation between the results for MMP-9 activation ratio obtained by zymography and active MMP-9 by immunohistochemistry for 32 matched cases of tumor, we found a positive relationship between zymography results and immunohistochemical scores (rs = 0.584, *P* < 0.001).

### Detection of gelatinolytic activity by in situ zymography

All PTC tissues showed strong to moderate gelatinase activity in the follicular epithelium and, occasionally, in stromal cells (myo/fibroblast-like cells, endothelial cells, inflammatory cells, adipocytes). A duplicate tissue section incubated in the presence of 20 mM EDTA, a general inhibitor of MMP activity, exhibited almost no specific gelatinolytic activity. Normal thyroid follicular epithelial cells generally had negligible gelatinase activity. The difference between normal and cancerous tissues was obvious.

**Figure 5:**
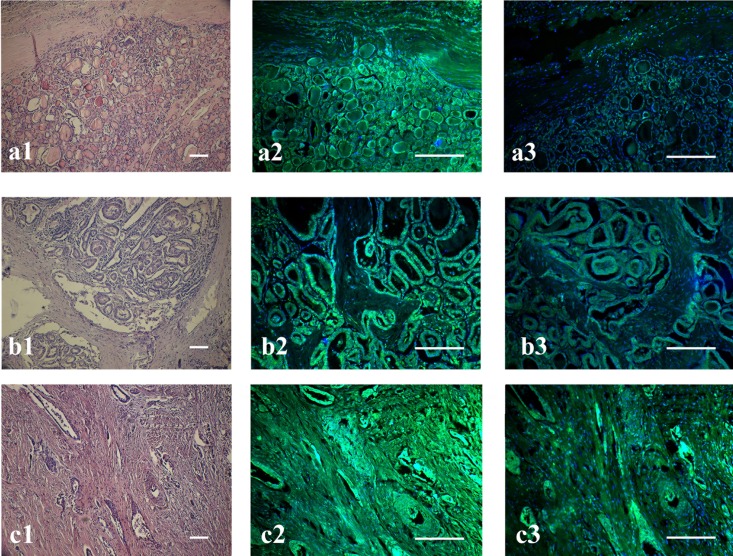
In situ zymography of gelatinase activity in papillary thyroid carcinomas with different degrees of neoplastic infiltration. (**a**) encapsulated carcinoma; (**b**) non-encapsulated tumor without thyroid capsule invasion; (**c**) tumor with extrathyroid extension. Horizontal layers (1-3) represent the consecutive sections of the same tumor. Left column, image of the hematoxylin/eosin stained papillary thyroid carcinoma (PTC) tissue sections. Middle column, distribution of gelatinolytic activity in serial tissue sections of the same areas of cases as the left column, subjected to fluorescent in situ zymography. Intense fluorescence was observed predominantly in the follicular epithelium, but also in some cells of tumoral stroma. Right column, matrix metalloproteinase-2 inhibitor-treated subsequent sections of the same cases as left. Decreased gelatinolytic activity is seen in thyrocytes and the stromal compartment.

In addition, we analyzed MMP-9 activity in PTC tissues showing various degrees of tumor infiltration. The PTCs exhibited prominent gelatinolytic activity, which was heterogeneous within a given tumor and did not differ substantially among different categories of tumor invasion (Figure 5 a2, b2, c2). Gelatinolytic activity was greatly diminished on sections incubated with 13.7 nM ARP-100, the MMP-2 inhibitor (Figure 5 a3, b3, c3), when compared with the control sections without inhibitor (Figure 5 a2, b2, c2). This indicates that ARP-100 at 13.7 nM can prevent MMP-2 activity, so almost all the breakdown of DQ gelatin may be attributed to the enzymatic activity of other gelatinolytic enzymes, ie, to MMP-9. After incubation with ARP-100, the level of green fluorescence compatible with gelatinolytic activity in encapsulated PTC (Figure 5, a3) was almost abolished. It may be concluded that gelatinase activity in most encapsulated PTCs mainly resulted from MMP-2, because activity was largely diminished by the inhibitor. It was also assumed that the remaining activity was predominantly due to MMP-9, which increased gradually in tumors with a greater degree of tumor infiltration (Figure 5, b3 and c3).

The results of in situ zymography were generally in correlation with previously used methods. The relative differences in fluorescence intensity observed by in situ zymography after applying ARP 100 in most cases showed the staining trend of active MMP-9 observed by immunohistochemistry and active to total MMP-9 ratio calculated by gel zymography. The discrepancies between results for in situ zymography and immunohistochemistry were confined to four cases, all belonging to groups A and B.

## Discussion

The study provides the first analysis of immunohistochemical expression of active MMP-9 in thyroid tissue sections and its relation to clinicopathological parameters of PTC. We showed that the production of total MMP-9 was greater in cancer than in the corresponding non-tumor thyroid tissue, unlike the report of Korem et al (18). In addition, our results revealed that total MMP-9 immunoreactivity was not correlated with any clinicopathological factor, which might suggest that mere overexpression of MMP-9 does not lead to more aggressive behavior of PTC. This is in contrast with previous reports (19,21,27,28), where abundant expression of MMP-9 correlated significantly with invasion and metastasis of thyroid carcinomas.

This study compared histological expression patterns of both total and active MMP-9 in PTC tissue samples in relation to some clinicopathological parameters. The novel finding was that active MMP-9 occurred almost only in cancer tissue and was correlated with age, presence of lymph node metastasis, extrathyroid invasion, and the degree of tumor infiltration, suggesting that MMP-9 was activated in tumor cells and that this was associated with aggressive tumor behavior in PTC.

To confirm the obtained immunohistochemical results of MMP-9 expression profiles and to find out whether there are any differences in the amount and degree of conversion of latent MMP-9 to its active counterpart between PTCs divided into distinct clinicopathological groups, we performed gelatin zymography. This procedure showed distinct bands of both latent and active MMP-9 in tumor extracts but vague bands in non-tumor extracts. The levels of active MMP-9 in our series of PTC tissue extracts were significantly higher in tumor tissues than in non-tumor tissues. To date, only the study of Maeta et al (19) analyzed the production and activation of MMP-9 in thyroid tissue by zymography, and both were greater in cancer tissue than in the corresponding non-tumor tissue. Our results extend these findings by demonstrating that there is not only more pro-active (latent) and active MMP-9 in PTC than in adjacent non-tumor tissue, but that the ratio of active to total MMP-9 is significantly elevated in PTC. Furthermore, this ratio increased substantially in tumors expressing a more aggressive phenotype, ie, in cases with extrathyroid invasion compared to those lacking these features. Patel et al (29) also reported that the activation ratio of MMP-9 was significantly elevated in malignant tissues compared to adjacent normal tissues in patients with oral squamous cell carcinoma, while the opposite was found for colorectal cancer and early-stage lung cancer, where the ratio was lower in tumor than in normal tissue (30,31).

Although gelatin zymographic analysis allows quantification of active and the pro-enzyme form of gelatinases, it does not provide information at the cellular level. This was overcome with the introduction of in situ zymography with the quenched fluorogenic substrate DQ-gelatin, which enables visualization of gelatinases activity in tissue sections. To our knowledge, in situ zymography on thyroid carcinoma tissue has been reported only by Nakamura et al (32). Unlike us, they used film in situ zymography method on frozen tissue sections, demonstrating that gelatinolytic activity was confined to follicular epithelial tumor cells. Our method employing DQ gelatin provided more detailed information on the localization of gelatinolytic activity, not only in follicular epithelial cells, but also within stroma, indicating that both compartments had the potential to play a role in biological behavior of PTC.

Finally, in order to more adequately define the source of gelatinolitic activity detected with DQ gelatin zymography on PTC tissue sections, we used a selective MMP-2 inhibitor. After selective inhibition of MMP-2 activity on a section of encapsulated PTC cases, the remaining gelatinase activity observed by in situ zymography was greatly diminished in most cases, suggesting a predominance of MMP-2 activity in these tumors, ie, almost an absence of MMP-9 activity. These results taken together with immunohistochemical staining of the same cancer areas for total and active MMP-9, suggested divergence between enzyme distribution and activity, either because MMP-9 is present mainly in its latent forms in most encapsulated PTCs, or because the active form is inhibited by endogenous antagonists. On the other hand, gelatinolitic activity was only slightly reduced by the MMP-2 inhibitor in tumors with the most advanced degree of infiltration, which may indicate a greater role of MMP-9 in these tumors. These cases also frequently exhibited a positive immunoreaction for active MMP-9 and efficient activation of MMP-9, as determined by gel zymography.

To conclude, the present study is the first report suggesting that evaluation of active MMP-9 by immunohistochemistry and the MMP-9 activation ratio by gelatin zymography may be a useful adjunct to known clinicopathological factors in predicting tumor behavior. Most important, in situ zymography with an MMP-2 inhibitor for the first time demonstrated a strong impact of MMP-9 activity on the degree of tumor infiltration during PTC progression.
